# Examining the operationalizability of findings from homicide investigations

**DOI:** 10.1192/j.eurpsy.2023.1870

**Published:** 2023-07-19

**Authors:** R. Nathan, R. Caplan, M. Gill-Mullarkey

**Affiliations:** 1 Research, Cheshire and Wirral Partnership NHS Foundation Trust; 2 Research, Cheshire and Wirral NHS Partnership NHS Trust, Chester; 3 Adult Social Care, Lancashire County Council, Preston, United Kingdom

## Abstract

**Introduction:**

Investigations into health and social care services offered to people who have died by suicide or who have committed a homicide have the potential to facilitate improvements in future practice. Such improvements are, however, dependent on the operationalizability of the recommendations of these investigations. Operationalizability in this context means the potential of the recommendations to alter the thinking/actions of practitioners involved in the areas of practice to which the recommendations relate. Critically, the proposed learning must make sense to practitioners in a multiplicity of single instance episodes of practice when the future is unknown. Although common content themes identified by investigations have been reported, no study has yet specifically examined how the framing of recommendations in investigation reports affects their operationalizability.

**Objectives:**

Primary objective: to pilot a novel approach to the thematic analysis of investigations into serious incidents which focuses on the operationalizability of recommendations for day-to-day practice. Secondary objective: to explore the operationalizability of the specific recommendations arising from a recent UK review of child homicides.

**Methods:**

A publicly available UK national review of child homicides by parents under social care services was subjected to a two-stage thematic analysis (firstly, to identify the types of thinking/acting that were scrutinised; and secondly to characterise the ways in which these thoughts/actions were appraised). The frame of reference for the thematic analysis was that of a practitioner involved in typical instances of practice where there is uncertainty about outcomes (i.e. real-life practice).

**Results:**

Stage 1 - Four types of thinking/acting were identified: (i) information gathering, (ii) interpretation, (iii) judgement formation, and (iv) decision to act (figure 1).Stage 2 - The thoughts/actions were appraised according to three key themes: (a) occurrence of thoughts/actions at a pivotal moment, (b) erroneousness of thoughts/actions, and (c) thoroughness of thoughts/actions.

**Image:**

**Figure:**
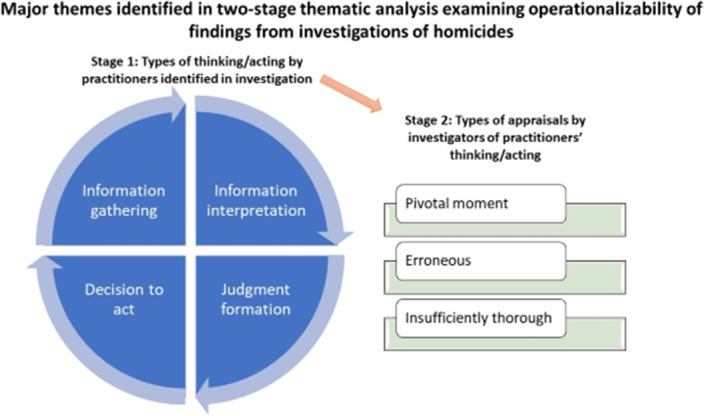

**Conclusions:**

With regard to the specific report analysed, the recommendations were found to have differing degrees of operationalizability. For instance, examples of ‘erroneous thinking/acting’ were more readily applicable to future practice (since they can be operationalised in terms of general principles). However, the notion of ‘pivotal moments’ is less useful, since the labelling of moments as ‘pivotal’ is dependent on a knowledge of the outcome and therefore would not have been readily identified contemporaneously in these cases (or, by extension in future similar cases prior to any serious incident). This pilot demonstrates that the novel approach used is a feasible way to examine not just the content, but also the utility, of investigation recommendations.

**Disclosure of Interest:**

None Declared

